# Understanding User Experience: Exploring Participants’ Messages With a Web-Based Behavioral Health Intervention for Adolescents With Chronic Pain

**DOI:** 10.2196/11756

**Published:** 2019-04-15

**Authors:** Annie T Chen, Aarti Swaminathan, William R Kearns, Nicole M Alberts, Emily F Law, Tonya M Palermo

**Affiliations:** 1 Department of Biomedical Informatics and Medical Education School of Medicine University of Washington Seattle, WA United States; 2 Department of Psychology St Jude Children's Research Hospital Memphis, TN United States; 3 Department of Anesthesiology and Pain Medicine School of Medicine University of Washington Seattle, WA United States; 4 Center for Child Health, Behavior and Development Seattle Children's Research Institute Seattle, WA United States

**Keywords:** data visualization, natural language processing, chronic pain, cluster analysis, technology

## Abstract

**Background:**

Delivery of behavioral health interventions on the internet offers many benefits, including accessibility, cost-effectiveness, convenience, and anonymity. In recent years, an increased number of internet interventions have been developed, targeting a range of conditions and behaviors, including depression, pain, anxiety, sleep disturbance, and eating disorders. Human support (coaching) is a common component of internet interventions that is intended to boost engagement; however, little is known about how participants interact with coaches and how this may relate to their experience with the intervention. By examining the data that participants produce during an intervention, we can characterize their interaction patterns and refine treatments to address different needs.

**Objective:**

In this study, we employed text mining and visual analytics techniques to analyze messages exchanged between coaches and participants in an internet-delivered pain management intervention for adolescents with chronic pain and their parents.

**Methods:**

We explored the main themes in coaches’ and participants’ messages using an automated textual analysis method, topic modeling. We then clustered participants’ messages to identify subgroups of participants with similar engagement patterns.

**Results:**

First, we performed topic modeling on coaches’ messages. The themes in coaches’ messages fell into 3 categories: Treatment Content, Administrative and Technical, and Rapport Building. Next, we employed topic modeling to identify topics from participants’ message histories. Similar to the coaches’ topics, these were subsumed under 3 high-level categories: Health Management and Treatment Content, Questions and Concerns, and Activities and Interests. Finally, the cluster analysis identified 4 clusters, each with a distinguishing characteristic: Assignment-Focused, Short Message Histories, Pain-Focused, and Activity-Focused. The name of each cluster exemplifies the main engagement patterns of that cluster.

**Conclusions:**

In this secondary data analysis, we demonstrated how automated text analysis techniques could be used to identify messages of interest, such as questions and concerns from users. In addition, we demonstrated how cluster analysis could be used to identify subgroups of individuals who share communication and engagement patterns, and in turn facilitate personalization of interventions for different subgroups of patients. This work makes 2 key methodological contributions. First, this study is innovative in its use of topic modeling to provide a rich characterization of the textual content produced by coaches and participants in an internet-delivered behavioral health intervention. Second, to our knowledge, this is the first example of the use of a visual analysis method to cluster participants and identify similar patterns of behavior based on intervention message content.

## Introduction

### Background

In recent years, an increased number of internet interventions have been developed in behavioral health, targeting a range of conditions and behaviors, including depression, pain, anxiety, substance use, sleep disturbance, psychotic disorders, and eating disorders. Delivery of interventions on the internet offers many benefits, including accessibility, cost-effectiveness, convenience, and anonymity [1,2]. However, though research has shown that internet behavioral interventions work, questions remain concerning how, why, and for whom, and there remains a need for better methods for investigating these questions [3-5]. There are gaps in our knowledge, including the need to identify predictors of therapeutic success or failure [6], reasons for attrition and dropout [7], long-term impacts, active intervention components, and methods for tailoring and promoting engagement [8-11]. Predicting which patients will respond best to which treatments [12] and personalizing interventions [13] are also important challenges. Finally, demonstrating treatment fidelity by showing that a treatment is applied consistently to all participants randomized to a treatment is critical [14].

Fortunately, with internet-delivered interventions, we are also seeing a diverse array of passively collected data which can help us better understand participants’ experiences. In this study, we are particularly concerned with textual data (eg, messages exchanged with a coach), which could, for example, provide insight into participants’ experiences and enable us to tailor interventions to their needs. Though it has been observed that there is potential to employ natural language processing techniques to personalize mental health treatments, to date, such examples are scarce [15].

Textual data could also help us learn more about the effects of coaching and intervention components. A few studies of adult populations have used content analysis to categorize and explore associations between coach behaviors (eg, task reinforcement and self-efficacy shaping) and treatment response [16-18]. Content analysis of client emails has also shown that attempts to try alternative behaviors and observation of positive consequences are associated with engagement, as measured by module completion [19,20]. Thus, developing better methods to examine textual data is important for a number of reasons, including personalization, assessment of the effectiveness of coaching, and understanding whether participants are learning and practicing the treatment content.

However, content analysis can be time-consuming and laborious. Some studies have used automatic methods of textual analysis, such as the Linguistic Inquiry and Word Count (LIWC; Pennebaker Conglomerates, Inc) software, to analyze communication patterns in therapeutic settings and online support groups [21-26]. The LIWC assesses word usage in particular domains such as positive and negative emotion, anxiety, and pronouns [27], but it does not facilitate thematic analysis. To better characterize the subject matter of communications, we could consider other automated forms of textual analysis, such as topic modeling [28] and document clustering [29,30], which have been used to evaluate health communication patterns in social networking platforms and online health communities, but not textual data from internet-delivered interventions.

### Objectives

In this study, we aimed to demonstrate the feasibility of a novel textual and visual analytic approach to identify patterns of engagement during an internet-delivered cognitive behavioral therapy (CBT) intervention for youth with chronic pain aged 11 to 17 years and their parents [31]. We have previously demonstrated the efficacy of an internet CBT intervention versus internet-delivered pain education on our primary outcome of adolescent activity limitations at 6-month follow-up [31]. In this secondary analysis, we focus only on youth randomized to the internet CBT arm because this was the only treatment arm in our trial that included human support. In the internet CBT intervention, participants could interact with a coach via an asynchronous message center. First, we present findings from topic modeling to characterize the primary themes of the messages sent by coaches and participants. The purpose of this step was to present an overview of the thematic content as well as to demonstrate how the results of topic modeling can differ depending on the content author. Second, we focus on intervention participants, employing cluster analysis to identify and visualize subgroups of participants with similar patterns of treatment engagement and message content.

Our work is novel in 2 ways: (1) in the use of topic modeling to provide a rich characterization of intervention participants’ experiences and (2) through the development of a visual method for comparing textual artifacts of participant engagement. We take a visual analytics approach, which facilitates interpretation of complex data by combining concepts from data mining, machine learning, human-computer interaction, and human cognition [32]. In health care, we often see it applied to electronic health records for the analysis of patient trajectories and to identify patients with similar clinical characteristics (eg, [33,34]). Extant literature has also included visual analysis of health-related communication [28,35,36], but to our knowledge, there has not been prior work using visual methods to examine coach-participant messages in internet interventions.

## Methods

### Internet-Delivered Cognitive Behavioral Therapy

**Figure 1:**
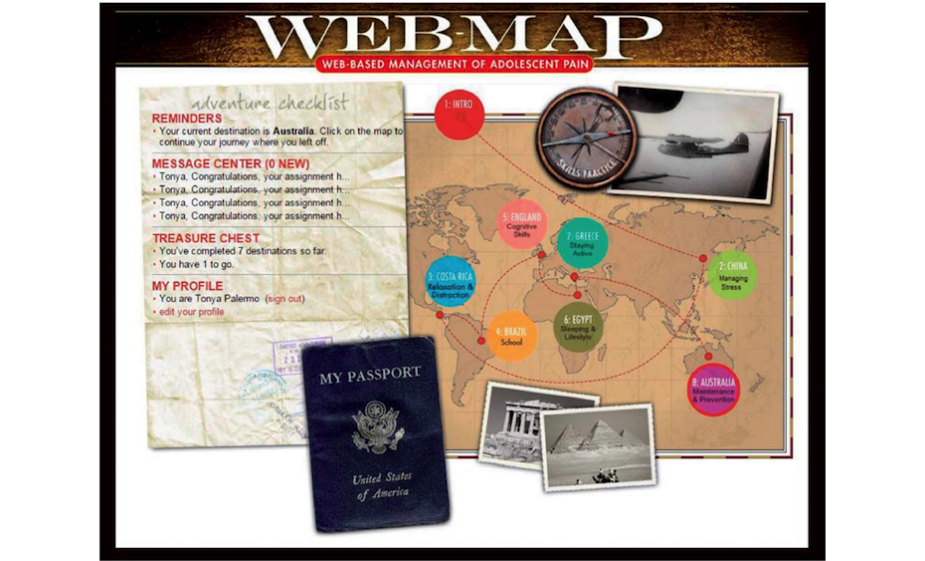
Web-based Management for Adolescent Pain (Web-MAP) homepage.

Adolescents and parents in the internet CBT condition received access to Web-based Management for Adolescent Pain (Web-MAP2), a pain self-management intervention based on cognitive-behavioral, social learning, and family systems theories. Treatment content and program features have been described in detail elsewhere [31] (see Figure 1 for a screenshot of the home page). The program has a travel theme with each treatment module representing a different destination from around the world. Adolescents and parents were provided with access to separate, password-protected websites that included treatment modules, audio files of relaxation exercises, a progress tracker, and a message center where they could exchange asynchronous messages with a coach.

Adolescent treatment modules included the following: (1) pain education, (2) recognizing stress and negative emotions, (3) relaxation methods, (4) coping with pain at school, (5) cognitive coping skills, (6) sleep hygiene and lifestyle skills, (7) increasing activity, and (8) relapse prevention. Parent treatment modules included the following: (1) pain education, (2) recognizing stress and negative emotions, (3) operant strategies I (attention and praise), (4) operant strategies II (reward systems and strategies to support school goals), (5) modeling, (6) sleep hygiene and lifestyle, (7) communication skills, and (8) relapse prevention. Adolescents and parents were asked to complete 1 treatment module per week, which was designed to be analogous to weekly sessions delivered in face-to-face CBT. In 6 of the 8 modules, adolescents and parents were asked to practice coping skills and complete weekly behavioral assignments related to their personalized goals.

In this secondary data analysis, we focused on understanding the content of messages exchanged between coaches and participants (adolescents and parents) in the message center. Through the message center, coaches provided participants with personalized feedback about each behavioral assignment. Adolescents and parents could also initiate messages to the coach via the message center at any time. During the trial, all assignments were reviewed by 1 of the 5 coaches with prior experience in CBT (4 psychology postdoctoral fellows and 1 masters-level therapist). Coaches responded to each behavioral assignment and all messages initiated by participants. Coaches used a manual to standardize all messages sent to participants, which emphasized rapport building (eg, “What do you like to do for fun?”), praise for skills practice (eg, “Great job spending more time in school this week!”), and strategies to overcome barriers to skills practice (eg, “Try practicing deep breathing at the same time every day.”). Coaches were supervised by a licensed clinical psychologist (TP) via regular message review to ensure adherence to the manual and standardization of messages sent to participants.

The study was approved by the primary site’s institutional review board and the institutional review boards at each referring center. Adolescents gave assent and parents provided informed consent before initiating any research procedures.

### Sample and Measures

The data for this study are from the internet-delivered CBT arm (n=138) of the trial (N=273) [31]. The inclusion criteria were as follows: (1) adolescent aged between 11 and 17 years, (2) adolescent reporting chronic idiopathic pain present over the previous 3 months, (3) adolescent reporting pain at least once per week, (4) parent reporting pain interfering with at least one area of daily functioning, and (5) the adolescent received a new patient evaluation in 1 of the participating pain clinics.

As this study focused on the messages sent by the coaches, teens, and parents, we excluded the participants who did not send any messages (n=15). In addition, 1 participant was excluded after the topic modeling and cluster analysis because they did not meet the eligibility criteria for the main study. We report the statistics for this sample (n=123).

At pretreatment, parents reported on adolescents’ age, gender, and racial background via the secure study website. Similar to other studies, we also measured engagement using unobtrusive measures of participant exposure and skills practice [11,37,38]. Specifically, we employed 4 measures of engagement: the number of messages sent, the average word count of participants’ messages, module completion rates, and percentage of interactive fields.

Interactive fields in all the modules facilitated personalization of the intervention. For example, in the module focused on parent operant training, parents were asked to input the behaviors they wanted to target in a behavior management plan; the behaviors that parents entered were subsequently used to remind parents what they want to focus on in the next week [39]. As completion of the interactive fields was not required, the percentage of interactive fields completed could be considered an indicator of the level of engagement.

### Using Topic Modeling to Characterize the Message Content

To identify topics discussed by coaches and participants, we employed a generative probabilistic modeling algorithm, Latent Dirichlet Allocation (LDA), to identify the main themes in the messages. LDA models documents as random mixtures over topics, where a topic is defined as a distribution of words [40]. The output of LDA is the distribution of topics within each document and a word distribution for each topic [41]. The keywords for each topic can then be used, along with documents that are predicted to have high proportions of the topics, to determine what the topics are. However, examining the documents along with their predicted probabilities for each topic is not necessarily an intuitive way to explore a document collection. Thus, in recent years, we have seen various efforts to create visual ways of exploring the topics (eg, [41-43]). In this section, we explain the details of the topic modeling procedure that we performed, and later, we will explain the visual application that we developed for exploring the textual data for internet-delivered interventions.

We used the LDA implementation available within the MAchine Learning for LanguagE Toolkit (MALLET) toolkit [44] to identify the most common topic within each message. We experimented with varying numbers of topics ranging from 10 to 45 and elected to use a 15-topic solution, which provided a balance between diversity of topics and ease of interpretability. Using a greater number of topics could lead to greater precision in terms of topic but could also make it more difficult for researchers to interpret.

When performing topic modeling, it is helpful to exclude words such as “a,” “an,” and “the” that appear frequently but add little meaning. To accomplish this, we employed the default stop word list that is provided with the MALLET toolkit, along with a custom stop word list consisting primarily of first names, to exclude coaches’ and participants’ names. We performed topic modeling separately on coaches’ and participants’ messages because there were marked differences in these messages, and combining them decreased topic coherence.

We verified topic assignments by randomly selecting 20 messages categorized under each topic and manually checking to see if the topic assignment was accurate. Where there were less than 20 messages pertaining to a topic, all messages assigned to that topic were verified. For participants, the accuracy rate was 76.5% (192/251). For coaches, the accuracy rate was 99.3% (298/300).

### Clustering and Visual Analysis of Participants’ Message Histories

We used cluster analysis to identify subgroups of participants that shared similarities in their message histories. Cluster analysis was performed using the Communication History Analysis Interface (CHAI), a visual interface that we developed which offers users the capability to visualize participants’ message histories, perform cluster analysis, and explore the results of cluster analysis.

To identify subgroups of participants with similar message histories, we employed the k-means clustering method [45] to cluster parent and teen pairs by the topics that they discussed with their coaches. K-means cluster analysis takes a set of n-dimensional points and clusters them into a set of K clusters [45]. Each parent and teen pair’s communications with the coaches were represented using a 15-dimensional vector, 1 dimension for each topic identified in the topic modeling procedure. To give an example, suppose a parent and teen pair authored 6 messages in total, 2 each for topics 3, 5, and 7. Their contribution would be represented by {0, 0, 2, 0, 2, 0, 2, 0, 0, 0, 0, 0, 0, 0, 0}. Thus, the vector representing each parent and teen pair would illustrate common topics within that pair’s communications, and the results of the cluster analysis would yield parent and teen pairs that discussed similar topics. As 1 topic, Time, was highly prevalent and yet had no specific meaning other than the references to time, it was excluded from the clustering.

We employed 2 methods together, visual examination and the inverse scree plot [46], to select the number of clusters. We plotted the variance for solutions with the number of clusters k varying from 1 to 20, and selected 4 as the optimal solution for 2 reasons. At this point the increasing the number of clusters led to less substantial decreases in variance, but there was not a “clear bend.” We visually examined solutions of differing numbers through the CHAI interface, deciding on 4 to err on the side of coarser clusters that illustrated differences in participants’ textual communications, but did not differentiate too granularly within the sample. The k-means clustering method can be susceptible to the starting seeds [47]. To avoid bias, we repeated the clustering with different starting seeds and observed that the defining characteristics of the clustering solutions remained the same in the repetitions.

**Figure 2:**
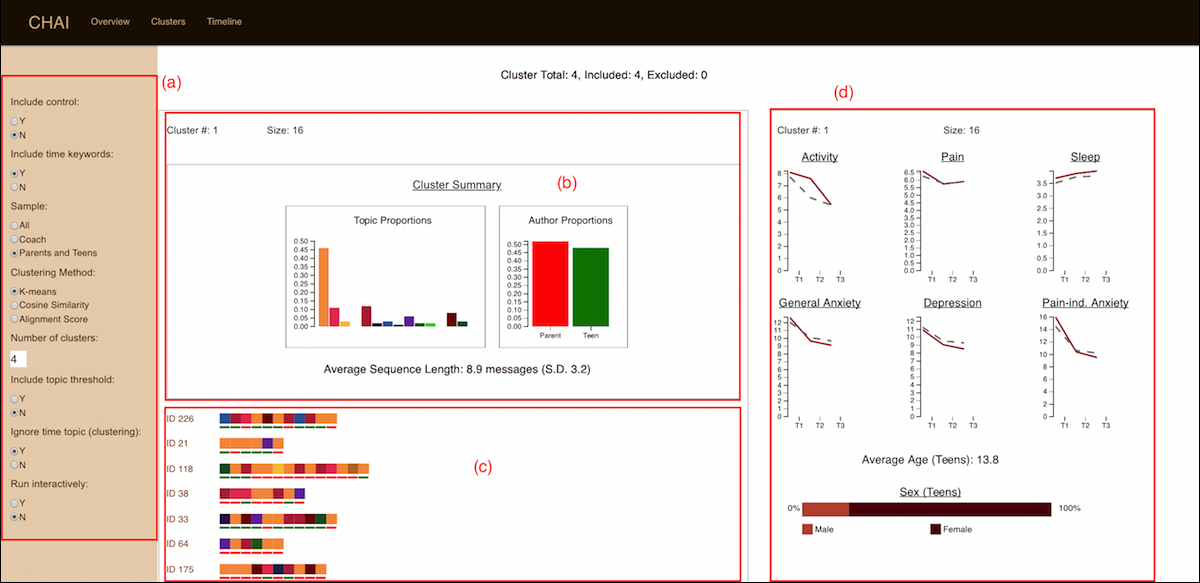
Communication history analysis interface: (a) cluster controls, (b) cluster engagement characteristics (theme proportions and parent/teen participation), (c) message sequences, and (d) cluster demographics.

We examined the results of the cluster analysis using the CHAI application that we developed. The clustering feature of this application features 2 primary views, an Overview of the clustering results (Figure 2) and a Cluster Detail view that can be used to examine the messages for each cluster. CHAI provides summaries of cluster engagement characteristics that show the prevalence of all topics in each cluster, so that users can compare the clusters in terms of topic and authorship. The CHAI application performs cluster analysis and displays participants’ message histories by cluster. For any given participant identification (ID) number, each message history is rendered as a horizontal sequence, with the earliest message to the left and the last message on the right. The right pane enables users to view outcomes and demographic characteristics for each cluster. The CHAI application was developed using Python, the machine learning library scikit-learn, and Web development frameworks and Javascript visualization libraries including AngularJS and D3.

### Characterizing the Clusters

To compare clusters, analysis of variance (ANOVA) is often used for interval/ratio variables and Chi-square analyses for categorical variables [48-50]. In this study, we employed a hybrid comparison method involving both visual analysis and statistical measures. A visual analysis of the message histories and engagement summaries for each cluster enabled us to characterize each cluster’s communication patterns in terms of topical emphasis. We then employed statistical measures to compare the clusters further. We performed ANOVA to compare the clusters in terms of the number of messages, message length, module completion rates, and percentage of interactive fields completed. We also compared the clusters on demographic characteristics using ANOVA for teen age and Fisher exact test for teen gender because of expected cell counts of less than 5 [51].

## Results

### Sample and Measures

Descriptive statistics for adolescents are presented in Table 1. The majority of the sample was female and white, with a mean age of 14.7 (SD 1.6) years. Overall, both parents and teens were highly engaged (Table 2). Descriptive statistics for the message data are presented in Table 3.

**Table 1:** Teen demographics (N=123).

Demographic characteristic		Statistics
Age (years), mean (SD)		14.7 (1.6)
**Teen gender, n (%)**		
	Male	26 (21.1)
	Female	97 (78.9)
**Teen racial background, n (%)**		
	White	115 (94.3)
	African American	2 (1.6)
	Native Hawaiian or other Pacific Islander	1 (0.8)
	American Indian or Alaskan Native	1 (0.8)
	Mixed	3 (2.5)

**Table 2:** Engagement measures.

Measure	Child, mean (SD)	Parent, mean (SD)
Modules completed	7.5 (1.3)	7.3 (1.5)
Percentage of interactive fields	79.6 (15.4)	72.8 (19.6)

**Table 3:** Message data.

Message type	Messages, n	Word count, mean (SD)
All messages	3426	132.7 (91.1)
By coaches	2692	149.8 (87.7)
By parents	347	88.6 (87.0)
By teens	387	52.7 (55.1)

### Using Topic Modeling to Characterize the Message Content

#### Primary Themes in the Coaches’ Message Content

We performed topic modeling and identified 15 topics. The themes in coaches’ messages fell into 3 categories: Treatment Content, Administrative and Technical, and Rapport Building (Table 4; Multimedia Appendix 1). These categories are consistent with those that we employed in our prior work [39].

**Figure 3:**
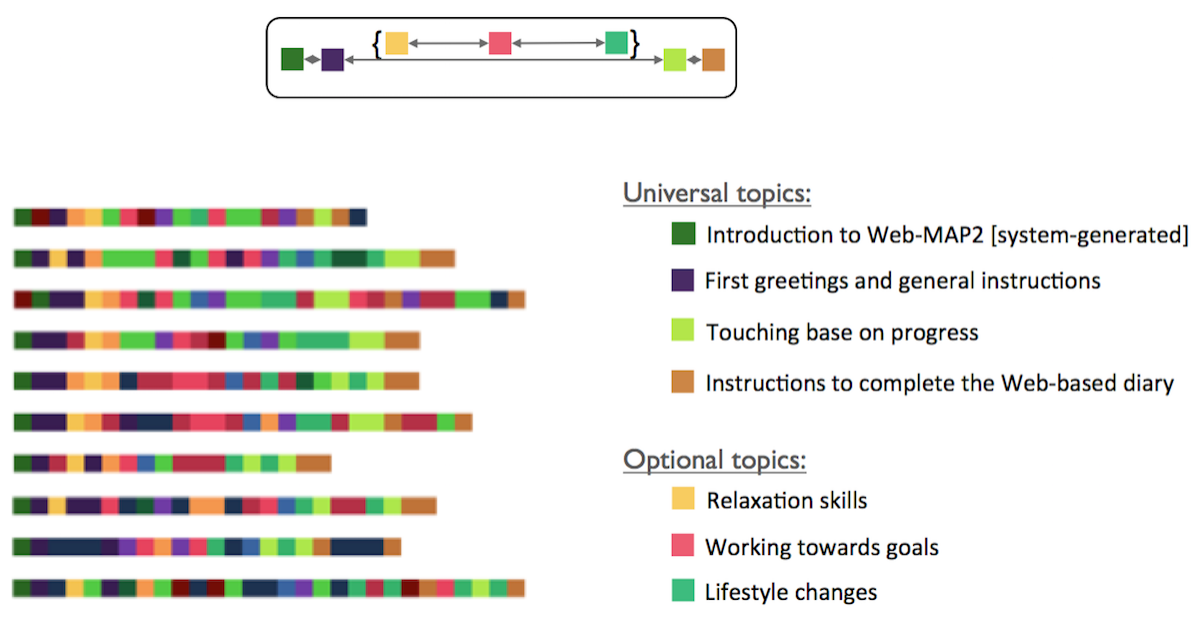
Temporal patterns in coaches’ messages. Web-MAP: Web-based Management for Adolescent Pain.

As expected, as the responses followed a coaching manual, there was a great deal of consistency in the topics and their order. In Figure 3, we see examples of coaches’ message histories, with each series corresponding to a different parent and teen. Certain topics appeared almost universally, such as the coach’s initial greeting in the beginning and the instructions to complete the Web-based diary at the end. Summation of progress and encouragement occurred toward the end of the intervention. Other topics such as relaxation skills, working toward goals, and lifestyle changes reflected the treatment content and followed the order of modules in Web-MAP2. This visualization can help us to verify that treatment is being delivered consistently to intervention participants.

##### Treatment Content

The main themes of the coaches’ messages reflected the treatment content. Some topics, such as Lifestyle Changes, complemented the skills that participants learned. Other topics, including guidance on how to use Web-MAP2 and touching base on participants’ progress, were not tied to particular treatment content:

It sounds like you're trying to change your eating habits which is a great idea. It can be hard to eat when you don't really feel hungry! Adding snacks throughout the day of food that used to be enjoyable to you is one strategy to help kick-start your appetite.

ID 190, in Lifestyle changes

I’ve looked at the progress tracker in your Passport and [participant name]’s pain sleep and ability to do things with friends have steadily improved since your family started Web-MAP2. That’s great! Congratulations to you both!

ID 286, in Touching base on progress

**Table 4:** Topics in coaches’ messages.

Topic number		Main theme	Keywords	Percentage^a^
**Treatment Content**				
	1	Reinforcing behaviors in parents	Great destination praise assignment congratulations move behaviors praising approved sounds job positive nice work staff negative	4.6
	2	Relaxation skills	Relaxation breathing practice practicing time deep feel bit find destination relaxing congratulations assignment skills approved good mp move audio	3.8
	3	Working toward goals	School plan work system great assignment goal destination reward approved move congratulations staff working sounds goals set time relaxation	8.9
	6	Encouraging parents to share their coping strategies	Strategies stress great coping helpful life stressed job approved part move types learn normal destination assignment congratulations staff work	4.3
	8	Thought replacement, thought stopping and relaxation techniques	Practice relaxation skills work great job day home practicing thoughts good thought exercises helpful assignment minutes mp ipod school	6.9
	10	Encouragement and strategies of how to utilize the program	Skills pain program work good learning staff hear great logging helpful find question week encourage manage sounds strategies time	7.4
	12	Lifestyle changes	Great sleep work lifestyle goal water habits making sounds goals set good congratulations assignment move approved destination make specific	8.7
	13	Touching base on progress	Program skills pain learned ability great passport participate activities progress continue tracker refresher return congratulations finished job looked anytime	8.2
**Administrative and Technical**				
	5	Instructions/reminders to complete the Web-based diary	Home staff question online section complete reminders back follow diary destination show directions visited link passed shown months pointing	10.3
	7	Responding to questions and/or information about assignments	Assignment program week complete staff questions message assessment destination days time touch log working send destinations approve back completed	7.5
	9	First greeting to participants and general instructions	Week destination assignment questions log complete clues program stress learning strategies approved working move find end things pain coach	11.2
	11	Introduction to Web-MAP2 and general instructions	Introduction journey home destination online complete final destinations questions begin staff start participation path proceed baby awhile camps parades	5.4
**Rapport Building**				
	4	Responding to participants’ descriptions of activities, interests, and family	Great fun sounds time nice work weekend staff logging family hear good friends awesome play week things busy message	8.0
	14	Expressing empathy, followed by constructive feedback	Time family teens feel pain good system important make start day behavior reward goals problem choose challenging things	3.2
	15	Asking for updates about life and general treatment progress	Note hear things drop update move assignment congratulations staff destination time approved great fun sounds messaging working system job	1.6

^a^Proportion of messages that were assigned to this topic.

##### Administrative and Technical

This category included the generic, fully scripted reminders or introductions that the system sent, the administrative messages that coaches sent (eg, self-introductions and reminders), and coaches’ response to administrative and technical questions:

Now that you've visited all of your Web-MAP2 destinations we'd like you to again complete your online diary.

ID 249, in Instructions/reminders to complete the Web-based diary

It might be helpful to read the Brazil readings today/tomorrow so you can think about the assignment over the week and submit the assignment this weekend.

ID 209, in Responses to questions and/or information about assignments

##### Rapport Building

Some topics focused on building rapport with participants, empathizing with them and providing support and guidance for incorporating the skills participants learned into their own lives. The position of these topics within a message sequence was unrelated to participants’ progress through the modules:

Thanks for sharing a little bit more about your family [participant name] it's great that you're all so active and enjoy sports. I'm really glad to hear that [participant name] has found a way to continue to be involved through coaching--when she is ready to play again it will make the transition a lot easier

ID 222, in Responses to participants’ descriptions of activities, interests, and family

#### Primary Themes in the Participants’ Message Content

We employed topic modeling to participants’ messages. Overall, 15 topics were identified, and similar to the coaches’ topics, these appeared to be subsumed under 3 high-level categories: Health Management and Treatment Content, Questions and Concerns, and Activities and Interests (Table 5; Multimedia Appendix 2). In addition, 2 topics, Family and Time, were reflections of common linguistic usage but not necessarily thematically important and will not be reviewed in detail in this section.

##### Health Management and Treatment Content

One of the main themes in participants’ messages was their health management and treatment content. Many of the messages illustrated how participants were incorporating the skills that they were learning in their own lives:

Its been a long road but finally [participant name] is feeling better. Her pain is now a 6 out of 10 when it was a 10 for the longest time. It definately helps to have the education and training that you have provided

ID 53, in Progress in learning pain and stress management techniques

hello: one way that we have changed is to let one another finish what is being said before jumping in: another way is the tone that is used: its a more calmer tone rather than a frustrated tone.

ID 15, in Rewards system, coping and achieving goals

##### Questions and Concerns

The Questions and Concerns category included 3 topics. Questions and Concerns about Assignments included updates that participants had completed assignments as well as inquiries about technical problems that they had regarding assignment completion, such as the system not storing that the assignment had been completed and asking participants to do the assignment again. The Suggestions topic included messages in which participants asked for guidance for a problem or made a suggestion about Web-MAP2. Finally, the Questions topic included a diverse range of questions, many regarding gift cards or technical errors:

I just wanted to let you know that I did answer the questions in this assignment at the beginning of the week. I wanted to reread some notes yesterday and when I went backwards in the lesson it must have reset my answers from earlier in the week.

ID 215, in References to assignments

I was wondering if you have any suggestions for ways that I could stay healthy during summer when my routine is not regular.

ID 74, in Suggestions

**Table 5:** Topics in participants’ messages.

Topic number		Themes	Keywords	Percentage
**Health Management and Treatment Content**				
	3	Progress in learning pain and stress management techniques	Pain time great program things good talk helpful learning techniques life support skills starting working level stress study dealing	11.8
	4	Pain	Pain school time day back work hard week days bad past program feel working put weeks started made problem	16.1
	5	Medications, nutrients and lab results	Prescribed day attention mg caffeine vitamin continued pill results symptoms November needed relief normal work bleeding progesterone bcp uti	0.5
	11	Rewards system, coping and achieving goals	System reward kids rewards privilege points worked motivated shows exams weekly sessions studying encouraged totally sign pretty talked fighting	3.3
	14	Fatigue, sleep, relaxation techniques	Sleep relaxation bed find breathing staying sleeping imagery helping practice asleep fatigue deep muscle hours helps night extremely told	4.6
**Questions and Concerns**				
	1	References to assignments	Back week complete assignment completed destination message brazil finished wanted log finish point logged diary destinations end costa thought	13.6
	2	Suggestions	Suggestions tq interested making things email walking process change putting small positive concerns important visit healthy medical challenge strategies	2.2
	8	Questions	Question make class teacher card place gift work continue found amazon email principal music special wondering privileges comfortable pm	3.8
**Activities and Interests**				
	15	Fun with family and friends	fun time friends play enjoy family day meet things nice great watch playing games soccer read pretty kids favorite	16.6
	6	Creative arts	Dog back show book mom walk picture sit dress pretty ups light continue black room hit front middle lab	3.3
	7	Music, sports, and school	Year years grade team school high people drive place plays called chorus trombone band telling funny students graduation glad	1.5
	10	Drama and reading	Drama schools rock club theatre reading show festival play England kohl hear bit excited players shows production piano props	2.4
	13	Trips	Home trip water softball july track crew cheer short till checking change fine feet vomiting sounds fruit taking grandma	1.9
**Other Topics**				
	9	Family	Year family husband children close home brothers call therapy lives told seattle blood older years thinks needed turned occasionally	2.2
	12	Time	Week school weekend good home back year today mom start happy wait work family assignment coming thing end break	16.6

##### Activities and Interests

A significant portion of participants’ messages described activities, interests and hobbies they enjoyed. The topics were primarily differentiated according to particular hobbies and interests, as specified by their corresponding keywords. These topics demonstrated ways in which their pain and other aspects of their health might interfere with their activities and how participants reacted:

I am into sports and other activity’s and when i am doing something I will start hurting but I don’t do anything about it because I will not let the pain stop me from doing what I am doing.

ID 104, in Fun with family and friends

### Clustering and Visual Analysis of Participants’ Message Histories

**Figure 4:**
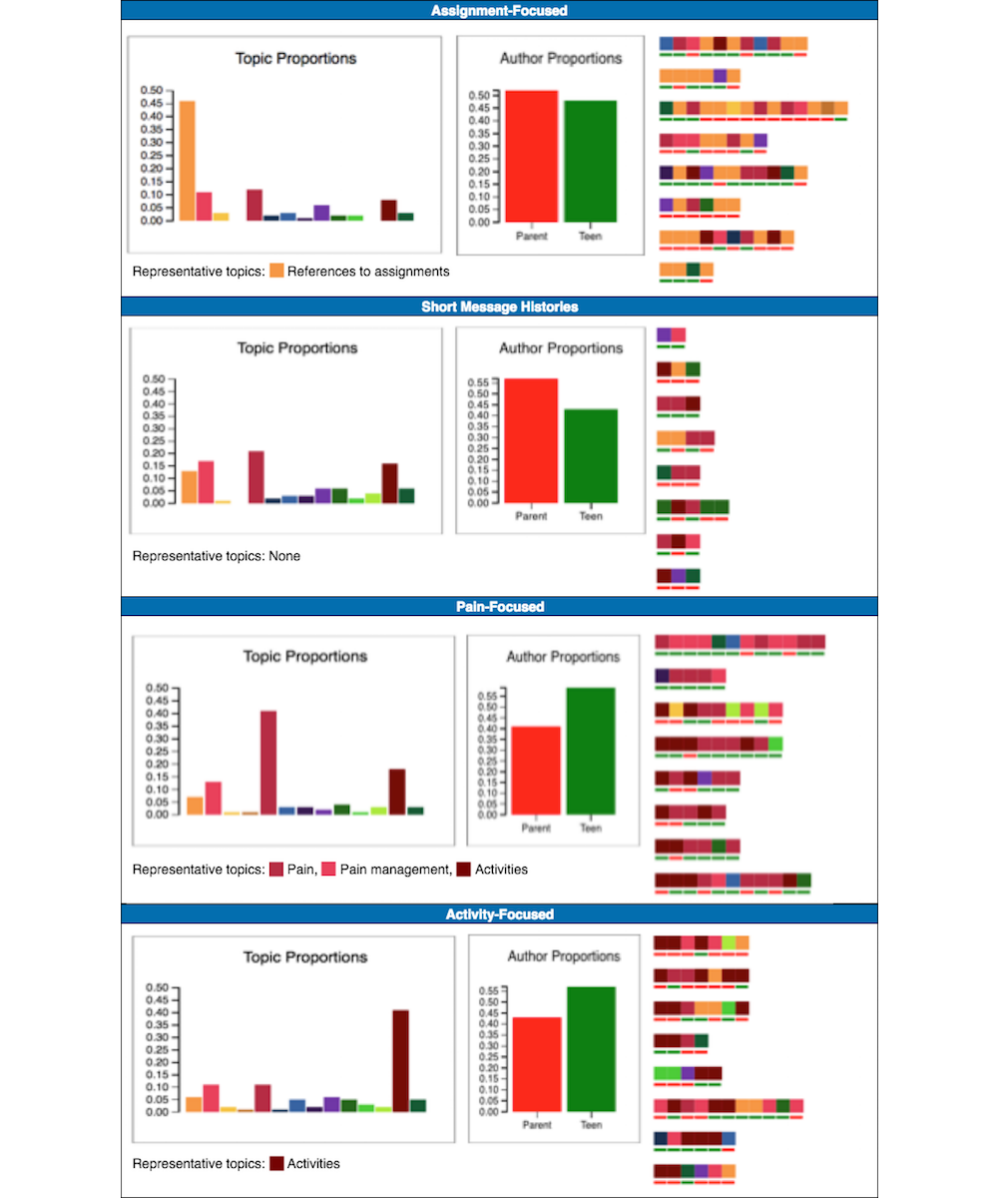
Topic proportions, parent-teen message proportions, and representative topics for each cluster. Red underline denotes a parent-authored message, and green underline denotes a teen-authored message.

We performed k-means clustering and selected 4 as the optimal clustering solution as described in the Methods section. Each of the clusters had a distinctive characteristic, either in terms of topic or extent of communication (Figure 4). Statistics for cluster membership and participation appear in Table 6.

#### Characterizing the Clusters

##### Assignment-Focused

The defining characteristic of this cluster was the prevalence of messages relating to assignments (Figure 4, orange). Participants were diligent about completing tasks and giving updates whenever they were delayed. Salient recurrent issues included technical problems and confusion about what they were supposed to be doing. With regard to the former, participants often reported that they had completed an assignment, but were asked to do the assignment again, leading to confusion and frustration. With regard to the latter, participants were sometimes generally confused about the program, but there were also more specific causes of confusion, for example, when lessons called for doing something related to school and school was not in session. Overall, there was a significant difference in age between the clusters (*F*_*3,119*_=3.1; *P*=.03). As the average age of the Assignment-Focused cluster was lower than the other groups, they perhaps needed more direction than the intervention participants in other clusters.

##### Short Message Histories

This cluster was the largest of the 4 and included approximately half of the sample. The distinguishing characteristic of this cluster was that there were significantly fewer messages sent as compared with the other clusters (Table 6), and there were no striking patterns in the topics discussed. Participants’ messages tended to be responses to questions from the coaches or to assignments. There were some technical questions, but otherwise, participants rarely reached out themselves to start a conversation or overtly ask for guidance. There were a fair number of apologetic utterances by participants, explaining that they had not spent time on Web-MAP2 because of other commitments.

##### Pain-Focused

In this cluster, participants’ messages focused primarily on pain and secondarily on pain management and activities (Figure 4, dark, light pink, and brown, respectively). Both parents and teens described the pain and other health issues that they experienced and the efforts that they made to deal with these issues. Their health status had a clear impact on their lives, in terms of their schedules and daily routines. Health care systems and health care providers also played a prominent role in conversations. Some of the messages in this cluster were long, with participants presenting detail concerning issues that they were having, reductions in their pain, or telling coaches about strategies that had had a positive effect.

##### Activity-Focused

A substantial part of the message content of this cluster was related to activities. These messages tended to have a more conversational feel, with participants sharing excitement and other sentiments toward the activities that they were engaged in, such as going to concerts, participating in drama, reading, and camping. There was also content concerning participants’ integration of the skills that they were learning. There were occasional questions of a relationship building nature, in which participants shared what they were doing, and then asked coaches questions about them, for example, what they had done over the weekend and what they liked to do. Like the Pain-Focused cluster, teens tended to engage with the coaches more than the parents.

#### Comparing Engagement Patterns Across Clusters

Aside from the number of messages, we investigated whether the clusters differed in terms of engagement through 3 other measures: message word counts, module completion rates, and percentage of interactive fields completed. The average message word count was not significantly different across clusters. For both the other types of engagement, the differences were significant at the .05 level for teens but not for parents (Table 6). Overall, the patterns across clusters were similar to what we observed with the number of messages. The parents and teens in the Short Message Histories cluster had the lowest module completion rates and interactive fields completed, with 1 exception: the parents in the Assignment-Focused cluster exhibited the lowest percentage of fields completed.

**Table 6:** Cluster membership and participation.

Comparison dimension		Assignment-Focused (n=16)	Short Message Histories (n=62)	Pain-Focused (n=20)	Activity-Focused (n=25)	*F* value (df)	*P* value
**Age (years), mean (SD)**							
	Teen	13.80 (1.4)^a^	15.01 (1.6)^b^	14.69 (1.7)	14.27 (1.6)	3.1 (3,119)	.03^c^
**Gender, observed/expected**							.93^d^
	Male	3/3	14/13	3/4	6/5	—^e^	—
	Female	13/13	48/49	17/16	19/20	—	—
**Number of messages, mean (SD)**							
	Teen	4.38 (3.5)^a^	1.65 (1.7)^b,f,g^	5.4 (2.8)^a^	4.24 (3.0)^a^	16.2 (3,119)	<.001^c^
	Parent	4.56 (3.0)^a^	1.95 (1.6)^b,f^	3.80 (2.7)^a^	3.04 (2.0)	8.7 (3,119)	<.001^c^
**Message word count, mean (SD)**							
	Teen	32.23 (1.0)	25.00 (1.9)	32.28 (1.0)	34.90 (0.4)	2.1 (3,119)	.10
	Parent	41.7 (1.0)	42.58 (1.9)	46.39 (1.1)	46.64 (0.4)	0.3 (3,119)	.80
**Module completion rate, mean (SD)**							
	Teen	7.63 (0.9)	7.13 (1.6)^g^	7.65 (0.8)	7.92 (0.4)^a^	2.8 (3,117)	.05^c^
	Parent	7.44 (1.0)	7.00 (1.9)	7.6 (1.1)	7.88 (0.4)	2.5 (3,117)	.06
**Interactive fields completed, mean (SD)**							
	Teen	82.73 (12.0)	75.18 (18.8)^g^	83.07 (8.5)	85.58 (7.4)^a^	3.8 (3,119)	.01^c^
	Parent	68.84 (17.3)	70.27 (22.5)	72.72 (19.5)	81.81 (7.6)	2.4 (3,119)	.07

^a^The given cluster is significantly different from the Short Message Histories cluster at the .05 level.

^b^The given cluster is significantly different from the Assignment-Focused cluster at the .05 level.

^c^Significant at the .05 level.

^d^Fisher’s Exact Test was performed because the gender variable is categorical and had expected cell values of less than 5 (please refer back to the Methods section for more detail).

^e^Not applicable.

^f^The given cluster is significantly different from the Pain-Focused cluster at the .05 level.

^g^The given cluster is significantly different from the Activity-Focused cluster at the .05 level.

## Discussion

### Principal Findings

In this secondary data analysis, we aimed to demonstrate the feasibility of employing a text and visual analytics approach to automatically characterize the intervention experience and identify subgroups of users with similar participation patterns in an internet-delivered behavioral health intervention. Our approach had 2 main parts. First, we employed automated text analysis methods to identify the primary themes of the messages sent by coaches as well as adolescent and parent users. Using a technique called topic modeling, we identified 15 topics from the coaches’ messages, which were subsumed under the high-level categories of Treatment Content, Administrative and Technical, and Rapport Building. We also examined the main themes that were discussed by adolescents and their parents, identifying 15 topics subsumed under 3 high-level categories: Health Management and Treatment Content, Questions and Concerns, and Activities.

Second, we employed k-means cluster analysis to identify subgroups of participants who shared similarities with one another in terms of their message content. The solution included 4 clusters: Assignment-Focused, Short Message Histories, Pain-Focused, and Activity-Focused. As compared with the other clusters, the Assignment-Focused cluster experienced proportionately greater issues and confusion with the program. Participants in the Short Message Histories cluster engaged significantly less with the coaches than the other clusters. The Pain-Focused and Activity-Focused clusters shared more with the coaches, with the thematic foci indicated in the cluster names.

**Figure 5:**
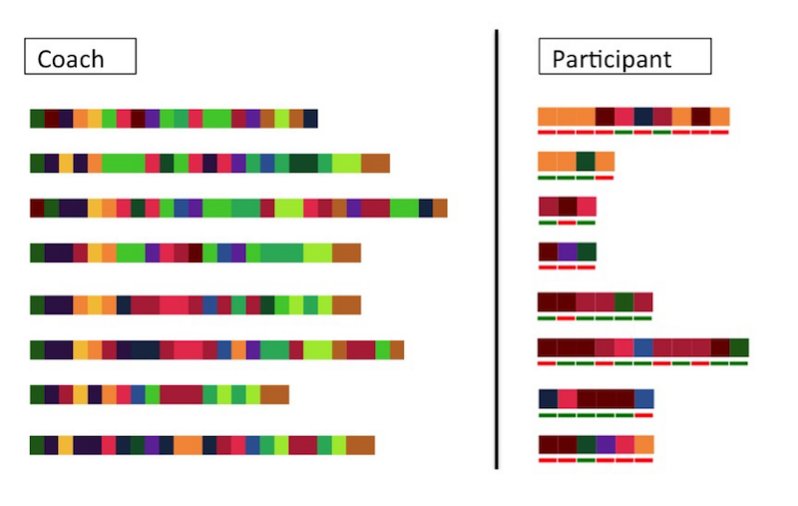
Temporality and the lack of temporality in coach versus participant sequences. Colored blocks denote messages and their assigned topics. Please see Multimedia Appendices 1 and 2 for color keys.

These clusters reflect different tendencies in the topics of conversation and interaction patterns within the sample. If cluster profiles could be formed in real time, participants’ interaction patterns and preferences could be considered in personalizing interventions. For example, if we are able to detect early on that certain participants need more feedback about assignments, coaches can offer additional guidance or the system can provide additional scaffolding for those participants. Whereas the coaches’ messages exhibited a clear temporal progression, the main message characteristics of the participant clusters demonstrated no such progression, suggesting that without additional guidance, engagement characteristics shown by participants are likely to remain consistent over time (Figure 5). If additional guidance or scaffolding were provided to these participants, we could then observe whether the thematic content of participants’ messages changes after the introduction of this additional support.

### Methodological Implications

We now consider the methodological contributions of the work presented. First, we demonstrated that topic modeling can produce coherent themes in textual data from internet-delivered interventions. The themes that were reported in the coaches’ messages were consistent with the topics covered in the Web-MAP2 modules, and the emergent topics reflected therapist skills, such as task reinforcement and encouragement, that have been reported in previous research on therapists’ behaviors [16-18]. This concordance suggests that topic modeling could potentially be used to assess treatment fidelity in internet-delivered behavioral health interventions as an alternative to qualitative content analysis methods that may be more time-consuming and as a way to categorize data in a format that could be used in subsequent systematic analyses, such as the cluster analysis described in this study.

However, despite the promise that has been shown in the use of topic modeling as a technique for facilitating the analysis of textual data from internet-delivered interventions, the study also identified areas for improvement. The accuracy of the topic modeling algorithm on the participants’ messages was not as high as on the coaches’ messages. There are perhaps several reasons for this. First, as the coaches were expected to deliver the same treatment to all participants, the consistency of the messages led to better performance of the algorithm. In addition, the number of coaches’ messages was substantially greater than the number of participants’ messages. There is a need to consider ways to improve the performance of the topic modeling of patient messages. One possibility might be to employ a method that incorporates domain knowledge about what types of topics that we expect, such as seeded LDA [52].

A second major contribution of this work is the development of a visual method for depicting and comparing sequential textual data from intervention participants. The visual representation that we employed in this study facilitates quick identification of temporal characteristics of message data as well as comparison of message sequences. Moreover, the auxiliary visualizations (topic proportion and author proportion) that we developed facilitate characterization of cluster members’ engagement with the intervention through their textual contributions. This is a significant contribution to the body of research concerning visualization of temporal health care data, which has often focused on visualizing structured data from electronic health records [53], though there are a few examples of visualization of conversational data collected in clinical settings (eg, [36,54]).

The work that we present here also suggests possibilities for just-in-time monitoring of behavioral health interventions. In the case of online forums, coaches or moderators often have a high volume of messages to monitor, and automated detection of messages of interest can reduce this burden [55]. Previous work has employed natural language processing methods to identify messages suggesting recovery problems in a substance abuse forum [55], and threads in an online diabetes community requiring moderator assistance [56].

Our results suggest that there is potential for the use of the techniques developed here to flag messages and issues for coaches to follow up on. In this study, the Questions and Concerns category was perhaps the one of greatest interest, as it included messages in which participants could benefit most from additional guidance. Automatically identifying these types of messages can provide insight into additional areas in which participants need information or support. Though preliminary, these results suggest that a dashboard could be developed that hides routine messages, identifies messages of interest, and categorizes and organizes issues for coaches to address. To increase the viability of such a dashboard, there is a need for additional work, involving input from intervention coaches, to ensure that the visual displays are clinically relevant and meaningful.

### Limitations and Future Directions

Our analysis has various limitations. First, we identified groups of individuals with shared characteristics in terms of content and volume of communication, measured through the number of messages sent. There may be a need for richer characterization of participant experiences for the purposes of tailoring and personalization. To do so, one might consider examining clustering solutions with a larger number of clusters or employing additional features in the cluster analysis to represent other dimensions of participant experience.

Second, in this study, we focused on the participants in our cluster analysis. In the future, we plan to develop visual methods to examine the dyadic interaction between coaches and participants. If we are able to identify frequent patterns of interaction and their consequences, then this information could help us to better understand how to provide support and guidance to participants during the course of an intervention. In our cluster analysis, we did not consider the timing of the topics in participants’ trajectories. In future work, it could be helpful to combine the temporality of messages, as well as other types of participant actions, in cluster analysis. Finally, we did not examine whether the patterns of interaction relate to treatment outcomes; future research is needed to understand the potential impact of interaction patterns on treatment benefit from internet-delivered interventions.

### Conclusions

In this study, we combined text and visual analytics techniques to explore messages authored in an internet-delivered behavioral health intervention for adolescents with chronic pain and their parents. We employed topic modeling to identify the main topics discussed by coaches and participants. Doing so helped us to characterize coaches’ behaviors and important aspects of participants’ experiences. Using cluster analysis and visual analytics, we identified participants who shared similarities in the ways that they interacted with coaches during the intervention. To our knowledge, this is the first example of the use of a visual analysis method employing textual data collected from an internet-delivered behavioral health intervention to cluster participants and identify similar patterns of behavior. Taking the entirety of participants’ engagement patterns—their topics of discussion, information needs, and interaction patterns into consideration—could potentially facilitate personalization and tailoring of interventions.

## Appendix

Multimedia Appendix 1 Topics in coaches’ messages.

Multimedia Appendix 2 Topics in participants' messages.

## Abbreviations

ANOVA: analysis of variance
CBT: cognitive behavioral therapy
CHAI: Communication History Analysis Interface
LDA: Latent Dirichlet Allocation
LIWC: Linguistic Inquiry and Word Count
Web-MAP2: Web-based Management for Adolescent Pain

## References

[1] Mohr DC, Burns MN, Schueller SM, Clarke G, Klinkman M (2013). Behavioral intervention technologies: evidence review and recommendations for future research in mental health. Gen Hosp Psychiatry.

[2] Palmqvist B, Carlbring P, Andersson G (2007). Internet-delivered treatments with or without therapist input: does the therapist factor have implications for efficacy and cost?. Expert Rev Pharmacoecon Outcomes Res.

[3] Klasnja P, Consolvo S, Pratt W (2011). How to evaluate technologies for health behavior change in HCI research. Proceedings of the SIGCHI Conference on Human Factors in Computing Systems.

[4] Schueller SM, Munoz RF, Mohr DC (2013). Realizing the potential of behavioral intervention technologies. Curr Dir Psychol Sci.

[5] Andersson G, Carlbring P, Berger T, Almlöv J, Cuijpers P (2009). What makes internet therapy work?. Cogn Behav Ther.

[6] Baumeister H, Reichler L, Munzinger M, Lin J (2014). The impact of guidance on Internet-based mental health interventions — a systematic review. Internet Interv.

[7] Riper H, Andersson G, Christensen H, Cuijpers P, Lange A, Eysenbach G (2010). Theme issue on e-mental health: a growing field in internet research. J Med Internet Res.

[8] Cunningham JA, Gulliver A, Farrer L, Bennett K, Carron-Arthur B (2014). Internet interventions for mental health and addictions: current findings and future directions. Curr Psychiatry Rep.

[9] Knowles SE, Toms G, Sanders C, Bee P, Lovell K, Rennick-Egglestone S, Coyle D, Kennedy CM, Littlewood E, Kessler D, Gilbody S, Bower P (2014). Qualitative meta-synthesis of user experience of computerised therapy for depression and anxiety. PLoS One.

[10] Short CE, Rebar AR, Plotnikoff RC, Vandelanotte C (2015). Designing engaging online behaviour change interventions: a proposed model of user engagement. Eur Health Psychol.

[11] Yardley L, Spring BJ, Riper H, Morrison LG, Crane DH, Curtis K, Merchant GC, Naughton F, Blandford A (2016). Understanding and promoting effective engagement with digital behavior change interventions. Am J Prev Med.

[12] Bagby RM, Quilty LC, Segal ZV, McBride CC, Kennedy SH, Costa JP (2008). Personality and differential treatment response in major depression: a randomized controlled trial comparing cognitive-behavioural therapy and pharmacotherapy. Can J Psychiatry.

[13] Ng MY, Weisz JR (2016). Annual Research Review: building a science of personalized intervention for youth mental health. J Child Psychol Psychiatry.

[14] Resnick B, Bellg AJ, Borrelli B, Defrancesco C, Breger R, Hecht J, Sharp DL, Levesque C, Orwig D, Ernst D, Ogedegbe G, Czajkowski S (2005). Examples of implementation and evaluation of treatment fidelity in the BCC studies: where we are and where we need to go. Ann Behav Med.

[15] Calvo RA, Milne DN, Hussain MS, Christensen H (2017). Natural language processing in mental health applications using non-clinical texts. Nat Lang Eng.

[16] Paxling B, Lundgren S, Norman A, Almlöv J, Carlbring P, Cuijpers P, Andersson G (2013). Therapist behaviours in internet-delivered cognitive behaviour therapy: analyses of e-mail correspondence in the treatment of generalized anxiety disorder. Behav Cogn Psychother.

[17] Holländare F, Gustafsson SA, Berglind M, Grape F, Carlbring P, Andersson G, Hadjistavropoulos H, Tillfors M (2016). Therapist behaviours in internet-based cognitive behaviour therapy (ICBT) for depressive symptoms. Internet Interv.

[18] Schneider LH, Hadjistavropoulos HD, Faller YN (2016). Internet-delivered cognitive behaviour therapy for depressive symptoms: an exploratory examination of therapist behaviours and their relationship to outcome and therapeutic alliance. Behav Cogn Psychother.

[19] Svartvatten N, Segerlund M, Dennhag I, Andersson G, Carlbring P (2015). A content analysis of client e-mails in guided internet-based cognitive behavior therapy for depression. Internet Interv.

[20] Soucy JN, Hadjistavropoulos HD, Couture CA, Owens VA, Dear BF, Titov N (2018). Content of client emails in internet-delivered cognitive behaviour therapy: a comparison between two trials and relationship to client outcome. Internet Interv.

[21] Dirkse D, Hadjistavropoulos HD, Hesser H, Barak A (2015). Linguistic analysis of communication in therapist-assisted internet-delivered cognitive behavior therapy for generalized anxiety disorder. Cogn Behav Ther.

[22] Owen JE, Yarbrough EJ, Vaga A, Tucker DC (2003). Investigation of the effects of gender and preparation on quality of communication in Internet support groups. Comput Hum Behav.

[23] van der Zanden R, Curie K, Van Londen M, Kramer J, Steen G, Cuijpers P (2014). Web-based depression treatment: associations of clients' word use with adherence and outcome. J Affect Disord.

[24] Siriaraya P, Tang C, Ang CS, Pfeil U, Zaphiris P (2011). A comparison of empathic communication pattern for teenagers and older people in online support communities. Behav Inf Technol.

[25] Lieberman MA, Goldstein BA (2006). Not all negative emotions are equal: the role of emotional expression in online support groups for women with breast cancer. Psychooncology.

[26] Kramer AD, Fussell SR, Setlock LD (2004). Text analysis as a tool for analyzing conversation in online support groups. CHI '04 Extended Abstracts on Human Factors in Computing Systems.

[27] Pennebaker JW, Boyd RL, Jordan K, Blackburn K (2015). The University of Texas at Austin.

[28] Chen AT, Zhu S, Conway M (2015). What online communities can tell us about electronic cigarettes and hookah use: a study using text mining and visualization techniques. J Med Internet Res.

[29] Lu Y, Zhang P, Liu J, Li J, Deng S (2013). Health-related hot topic detection in online communities using text clustering. PLoS One.

[30] Chen AT (2012). Exploring online support spaces: using cluster analysis to examine breast cancer, diabetes and fibromyalgia support groups. Patient Educ Couns.

[31] Palermo TM, Law EF, Fales J, Bromberg MH, Jessen-Fiddick T, Tai G (2016). Internet-delivered cognitive-behavioral treatment for adolescents with chronic pain and their parents: a randomized controlled multicenter trial. Pain.

[32] Caban JJ, Gotz D (2015). Visual analytics in healthcare--opportunities and research challenges. J Am Med Inform Assoc.

[33] Wang TD, Wongsuphasawat K, Plaisant C, Shneiderman B (2011). Extracting insights from electronic health records: case studies, a visual analytics process model, and design recommendations. J Med Syst.

[34] Gotz D, Sun J, Cao N, Ebadollahi S (2011). Visual cluster analysis in support of clinical decision intelligence. AMIA Annu Symp Proc.

[35] MacLean D, Hangal S (2009). You Didn't Tell Me That! Visualizing the Hidden Attributes of Online Health Communities.

[36] Angus D, Watson B, Smith A, Gallois C, Wiles J (2012). Visualising conversation structure across time: insights into effective doctor-patient consultations. PLoS One.

[37] Danaher BG, Seeley JR (2009). Methodological issues in research on web-based behavioral interventions. Ann Behav Med.

[38] Doherty G, Coyle D, Sharry J (2012). Engagement with Online Mental Health Interventions: An Exploratory Clinical Study of a Treatment for Depression. Proceedings of the SIGCHI Conference on Human Factors in Computing Systems.

[39] Law EF, Murphy LK, Palermo TM (2012). Evaluating treatment participation in an internet-based behavioral intervention for pediatric chronic pain. J Pediatr Psychol.

[40] Blei DM, Ng AY, Jordan MI (2003). Latent dirichlet allocation. J Mach Learn Res.

[41] Ganesan A, Brantley K, Pan S, Chen J (2015). arXiv.

[42] Chaney AJ, Blei DM (2012). Visualizing Topic Models. http://www.cs.columbia.edu/~blei/papers/ChaneyBlei2012.pdf.

[43] Chuang J, Manning CD, Heer J (2012). Termite: visualization techniques for assessing textual topic models. Proceedings of the International Working Conference on Advanced Visual Interfaces.

[44] McCallum AK (2002). MAchine Learning for LanguagE Toolkit.

[45] Jain AK (2010). Data clustering: 50 years beyond K-means. Pattern Recognit Lett.

[46] Lathrop RG, Williams JE (1987). The reliability of inverse scree tests for cluster analysis. Educ Psychol Meas.

[47] Khan SS, Ahmad A (2004). Cluster center initialization algorithm for K-means clustering. Pattern Recognit Lett.

[48] Yukselturk E, Top E (2012). Exploring the link among entry characteristics, participation behaviors and course outcomes of online learners: an examination of learner profile using cluster analysis. Br J Educ Technol.

[49] Ares G, Gámbaro A (2007). Influence of gender, age and motives underlying food choice on perceived healthiness and willingness to try functional foods. Appetite.

[50] Fotopoulos C, Krystallis A, Vassallo M, Pagiaslis A (2009). Food Choice Questionnaire (FCQ) revisited. Suggestions for the development of an enhanced general food motivation model. Appetite.

[51] McDonald JH (2009). Handbook of Biological Statistics.

[52] Ramesh A, Goldwasser D, Huang B, Daume H, Getoor L (2014). Understanding MOOC discussion forums using seeded LDA.

[53] Rind A, Wang TD, Aigner W, Miksch S, Wongsuphasawat K, Plaisant CC, Shneiderman B (2011). Interactive information visualization to explore and query electronic health records. Found Trend Hum–Comput Interact.

[54] Bartels J, Rodenbach R, Ciesinski K, Gramling R, Fiscella K, Epstein R (2016). Eloquent silences: a musical and lexical analysis of conversation between oncologists and their patients. Patient Educ Couns.

[55] Kornfield R, Sarma PK, Shah DV, McTavish F, Landucci G, Pe-Romashko K, Gustafson DH (2018). Detecting recovery problems just in time: application of automated linguistic analysis and supervised machine learning to an online substance abuse forum. J Med Internet Res.

[56] Huh J, Yetisgen-Yildiz M, Pratt W (2013). Text classification for assisting moderators in online health communities. J Biomed Inform.

