# Supplementary material for: Understanding User Experience: Exploring Participants’ Messages With a Web-Based Behavioral Health Intervention for Adolescents With Chronic Pain
Source: J Med Internet Res. 2019 Apr 15;21(4):e11756. doi: 10.2196/11756 (PMC6487347; doi:10.2196/11756)
Supplement: Multimedia Appendix 1 [file jmir_v21i4e11756_app1.pdf]

Multimedia Appendix 1. Topics in Coaches' Messages

Topic No.	Color	Main theme	Keywords	%[a]
**Treatment Content**				
1		Reinforcing behaviors in parents	great destination praise assignment congratulations move behaviors praising approved sounds job positive nice work staff negative	4.6
2		Relaxation skills	Relaxation breathing practice practicing time deep feel bit find destination relaxing congratulations assignment skills approved good mp move audio	3.8
3		Working towards goals	School plan work system great assignment goal destination reward approved move congratulations staff working sounds goals set time relaxation	8.9
6		Encouraging parents to share their coping strategies	Strategies stress great coping helpful life stressed job approved part move types learn normal destination assignment congratulations staff work	4.3
8		Thought replacement, thought stopping and relaxation techniques	Practice relaxation skills work great job day home practicing thoughts good thought exercises helpful assignment minutes mp ipod school	6.9
10		Encouragement and strategies of how to utilize the program	Skills pain program work good learning staff hear great logging helpful find question week encourage manage sounds strategies time	7.4
12		Lifestyle changes	Great sleep work lifestyle goal water habits making sounds goals set good congratulations assignment move approved destination make specific	8.7
13		Touching base on progress	Program skills pain learned ability great passport participate activities progress continue tracker refresher return congratulations finished job looked anytime	8.2
**Administrative/Technical**				
5		Instructions/reminders to complete the online diary	Home staff question online section complete reminders back follow diary destination show directions visited link passed shown months pointing	10.3
7		Responding to questions and/or information about assignments	Assignment program week complete staff questions message assessment destination days time touch log working send destinations approve back completed	7.5
9		First greeting to participants and general instructions	Week destination assignment questions log complete clues program stress learning strategies approved working move find end things pain coach	11.2
11		Introduction to Web-MAP2 and general instructions	Introduction journey home destination online complete final destinations questions begin staff start participation path proceed baby awhile camps parades	5.4
**Rapport Building**				
4		Responding to participants' descriptions of activities, interests, and family	Great fun sounds time nice work weekend staff logging family hear good friends awesome play week things busy message	8.0

Topic No.	Color	Main theme	Keywords	%[a]
14		Expressing empathy, followed by constructive feedback	Time family teens feel pain good system important make start day behavior reward goals problem choose challenging things	3.2
15		Asking for updates about life and general treatment progress	Note hear things drop update move assignment congratulations staff destination time approved great fun sounds messaging working system job	1.6

[a]Proportion of messages that was assigned to this topic
