# Supplementary material for: Understanding User Experience: Exploring Participants’ Messages With a Web-Based Behavioral Health Intervention for Adolescents With Chronic Pain
Source: J Med Internet Res. 2019 Apr 15;21(4):e11756. doi: 10.2196/11756 (PMC6487347; doi:10.2196/11756)
Supplement: Multimedia Appendix 2 [file jmir_v21i4e11756_app2.pdf]

Multimedia Appendix 2. Topics in Participants' Messages

Topic No.	Topic Color	Themes	Keywords	%
**Health Management and Treatment Content**				
3		Progress in learning pain and stress management techniques	Pain time great program things good talk helpful learning techniques life support skills starting working level stress study dealing	11.8
4		Pain	Pain school time day back work hard week days bad past program feel working put weeks started made problem	16.1
5		Medications, nutrients and lab results	Prescribed day attention mg caffeine vitamin continued pill results symptoms November needed relief normal work bleeding progesterone bcp uti	0.5
11		Rewards system, coping and achieving goals	System reward kids rewards privilege points worked motivated shows exams weekly sessions studying encouraged totally sign pretty talked fighting	3.3
14		Fatigue, sleep, relaxation techniques	Sleep relaxation bed find breathing staying sleeping imagery helping practice asleep fatigue deep muscle hours helps night extremely told	4.6
**Questions and Concerns**				
1		References to assignments	Back week complete assignment completed destination message brazil finished wanted log finish point logged diary destinations end costa thought	13.6
2		Suggestions	Suggestions tq interested making things email walking process change putting small positive concerns important visit healthy medical challenge strategies	2.2
8		Questions	Question make class teacher card place gift work continue found amazon email principal music special wondering privileges comfortable pm	3.8
**Activities & Interests**				
15		Fun with family and friends	fun time friends play enjoy family day meet things nice great watch playing games soccer read pretty kids favorite	16.6
6		Creative arts	Dog back show book mom walk picture sit dress pretty ups light continue black room hit front middle lab	3.3
7		Music, sports, and school	Year years grade team school high people drive place plays called chorus trombone band telling funny students graduation glad	1.5

Topic No.	Topic Color	Themes	Keywords	%
10		Drama and reading	Drama schools rock club theatre reading show festival play England kohl hear bit excited players shows production piano props	2.4
13		Trips	Home trip water softball july track crew cheer short till checking change fine feet vomiting sounds fruit taking grandma	1.9
**Other Topics**				
9		Family	Year family husband children close home brothers call therapy lives told seattle blood older years thinks needed turned occasionally	2.2
12		Time	Week school weekend good home back year today mom start happy wait work family assignment coming thing end break	16.6
